# Aberrant accumulation of Dickkopf 4 promotes tumor progression via forming the immune suppressive microenvironment in gastrointestinal stromal tumor

**DOI:** 10.1002/cam4.2437

**Published:** 2019-07-29

**Authors:** Ming Wang, Bo Ni, Chun Zhuang, Wen‐Yi Zhao, Lin Tu, Xin‐Li Ma, Lin‐Xi Yang, Zhi‐Gang Zhang, Hui Cao

**Affiliations:** ^1^ Department of Gastrointestinal Surgery, Renji Hospital Shanghai Jiaotong University School of Medicine Shanghai China; ^2^ State Key Laboratory of Oncogenes and Related Genes, Shanghai Cancer Institute, Ren Ji Hospital Shanghai Jiao Tong University School of Medicine Shanghai China

**Keywords:** DKK4, GIST, immune suppression, prognosis indicator, progression

## Abstract

**Background:**

Drug resistance and tumor recurrence are the major concerns in clinical practices of gastrointestinal stromal tumor (GIST), with the urgent requirement for exploring undiscovered pathways driving malignancy. To deal with these, recent studies have made many efforts to explore prognosis indicators and establish potential therapeutic targets.

**Methods:**

Expression profiles of different risks of GISTs were described and abundant clinical evidences supported our findings in this study. Following exploration in vitro by cell experiments and verification in vivo using tumor microarray were taken to elucidate the underlying mechanism, which drove the malignancy in GIST.

**Results:**

Dickkopf 4 (DKK4), as the canonical Wnt pathway antagonist, was unexpectedly and universally upregulated in high‐risk GISTs, and aberrant accumulation of DKK4 was closely correlated with poor prognosis. In addition, tumor‐derived DKK4 could decrease immune cells infiltration and activation in the tumor microenvironment, which decreased the antitumor effects in return. And this phenomenon was recurrent in human tumor specimens.

**Conclusions:**

Our findings identified DKK4 as a proper tumor biomarker for prognosis predicting and recurrence monitoring, and suggested a novel immune‐escape mechanism driving malignancy in GIST, which might be a potential therapeutic target to improve the effects of canonical RTK therapy and combined immunotherapy.

## 1 INTRODUCTION

Gastrointestinal stromal tumor (GIST) has aroused many concerns, as the most common mesenchymal tumor originating in gastrointestinal tract, accounting for 1%‐3% of all gastrointestinal malignancies.^1^, ^2^ Almost all cases of GISTs possess a gain function mutation of *KIT* or platelet‐derived growth factor receptor alpha (*PDGFRα*), followed by constitutive expression and ligand‐independent activation of its encoding protein‐receptor tyrosine kinase (RTK, KIT/PDGFRA), which results in continuous activation of oncogenic signaling.^3^, ^4^, ^5^, ^6^ Although RTK has been an excellent therapeutic target, significantly responsible to RTK inhibitors such as imatinib, there was almost no complete curative case, due to the inevitable drug resistance and tumor recurrence.^7^, ^8^, ^9^ GISTs have varying malignant potential with few reliable malignancy indicators, such as dystrophin,^10^ and KIT/PDGFRA are universally expressed in variable risks.^11^, ^12^, ^13^ In addition, the underlying mechanism of tumor malignant biology remains poor defined.^5^, ^14^ Overall, necessary and growing needs for credible biomarkers are proposed by recent studies and clinical practices, with the urgent requirement for exploring the undiscovered pathways driving GIST malignancy.

Wnt pathway is well‐known for contributing to tumor malignancy in multiple cancers,^15^, ^16^, ^17^, ^18^ which also serves as the pivotal mechanism driving tumor progression in GIST, announced by several previous evidences.^19^, ^20^ As a canonical Wnt pathway antagonist, DKK4 belonging to dickkopf (DKK) families is also transcriptionally activated by Wnt pathway transcriptional factor, forming an ideal regulatory loop.^21^, ^22^, ^23^, ^24^ Some earlier studies pointed that DDK4 drove the antitumor effects by antagonist of Wnt signaling.^25^, ^26^, ^27^ However, emerging evidence demonstrate that DKK4, upregulated by activated Wnt pathway, contributes to tumor malignancy in variable tumors, promoting tumor cells invasion and progression.^24^, ^28^, ^29^ These proofs suggest that DKK4 appears to develop multibiological function, which depends on its existing tumor microenvironment (TME) and the underlying mechanism of its targeting point. Overall, Wnt pathway serves as a malignancy‐driving mechanism in GIST and DKK4 is its canonical negative regulator and target‐gene coding protein. However, DKK4 remains weak understanding of its major function in GIST tumor progression.

Tumor‐infiltrating immune cells, such as CD8^+^ T cells, NK cells, and macrophages, populate in the GISTs, with the key role in tumor surveillance.^30^ In addition, these immune cells also contribute to the antitumor effects of RTK‐targeted therapy.^31^, ^32^ Immune microenvironment is a vital part of GIST microenvironment. Naturally, tumor cells will develop some mechanisms for confronting the strikes from the antitumor immune system, known as the canonical checkpoint regulating sites‐programed death 1 (PD1)/programed death ligand 1 (PD‐L1) axis and cytotoxic T lymphocyte‐associated antigen 4 (CTLA‐4) modulator.^32^, ^33^ Intriguingly, DKK family is also closely related to immune response and has the ability to block antitumor immune cell activation, mediating the tumor‐promoting mechanism.^34^

In this study, we discovered that tumor‐derived DKK4 is correlated with high‐risk stratification and poor prognosis of GIST patients. In addition, we present a new mechanism for tumor cells to evade immunological elimination, via secreting DKK4 to inactivate immune response. Our study supports that DKK4 is not only a good candidate for tumor marker, but also a potential therapeutic target to improve the effects of combined therapy.

## 2 MATERIALS AND METHODS

### 2.1 Patients and clinical specimens

All patients and clinical specimens involved in this study came from Renji Hospital, Shanghai Jiaotong University School of Medicine, while the specimen‐collecting process and study design were approved by the Renji Hospital Ethics Committee, with all patients’ informed consents. These specimens constitute four cohorts for different experimental design.

Cohort I, consisting of 132 cases of paraffin‐embedded tumor tissue, was collected from GIST patients who had undergone surgery at Renji Hospital from 2003 to 2010, arranged for tumor tissue microarray (TMA). Cohort Ⅱ was a group of eight GIST patients, containing four low‐risk and four high‐risk samples, stratified according to the postoperative pathological diagnosis. The eight patients accepted operations from January to July in 2010, with their tumor tissues storage at −80℃ for Expression Profile Analysis within 30 minutes since excised from body. In addition, 30 cases of fresh GIST tumor samples were collected from 2010 to 2012, designed for the extraction of mRNA and protein, which was defined as Cohort Ⅲ. To describe the profiles of individual serum concentration, preoperative plasma samples from 43 GIST patients, 20 healthy volunteers and 21 non‐GIST malignancies were collected from 2010 to 2012, among which 11 GIST cases were also collected for their bloods after surgery at intervals. These plasma‐originating individuals are defined as Cohort Ⅳ. All these patients were pathologically diagnosed by the Department of Pathology, Renji Hospital.

### 2.2 Cells and reagents

The human cell lines GIST‐882 and GIST‐430 were kindly gifted by Prof. Jonathan Fletcher (Brigham and Women's Hospital, Harvard Medical School), and GIST‐T1 was purchased from Cosmo Bio Co., Ltd. GIST‐T1 and 882 cells were maintained in RPMI1640 medium containing 10% fetal bovine serum (FBS) and 1% antibiotic mixture, while GIST‐430 cells were cultured in IMDM complete medium. All cells were incubated at 37℃ with 5% CO_2_, and all experiments of cells were conducted in biohazard safety equipment.

Recombinant DKK4 protein (1269‐DK) was purchased from R&D Systems, and administrated with 500 ng/mL for inhibiting immune cells migration and activation. Imatinib (HY‐15463) was provided by MedChemExpress.

### 2.3 GENT database

The Gene Expression across Normal and Tumor Tissue (GENT) database (http://medical-genome.kribb.re.kr/GENT/overview.php) is composed of publicly available and reliable data sets, involving 34 kinds of cancer types. It is a rare database including expression profiles of 66 cases of GIST samples, permitting to describe and compare the expression of DKK4 in variable tumors. Gene expression profile of every samples is also accessible to public, so we can use the original data for our correlation analysis.

### 2.4 Knockdown and overexpression of DKK4

For stable knockdown of DKK4 in GIST‐T1 cells, the lentivirus carrying short‐hairpin RNA (shRNA), DKK4‐Sh1, DKK4‐Sh2, and negative control (NC) was purchased from Genechem Co., Ltd. For stable overexpression of DKK4 in GIST‐430 cells, the lentivirus, LV‐DKK4 and NC were provided by Genechem Co., Ltd. With the density growing up to 50%‐60%, the lentivirus was incubated with cells in the presence of 1 × HitranasG transfection reagent (Genechem Co., Ltd). Stable knockdown and overexpression cells were maintained in complete RPMI1640 or IMDM medium with 2 mg/mL puromycin (Gibco). Subsequently, the transduction efficiency was detected in both mRNA and protein levels.

### 2.5 Real‐time PCR

Total RNA was extracted from fresh tumor tissues and GIST cells, using Trizol (Takara) according to its protocol, and then reversely transcribed to generate cDNA by High Capacity cDNA Reverse Transcription Kit (Takara). SYBR Green Master Mix (Takara), samples' cDNA, and primers (synthesized by Tsingke [Shanghai]) were mixed and then performed for real‐time PCR to detect the gene expression. Data were analyzed by the ΔΔCT values, using GAPDH as the internal reference. The primer sequences of DKK4 are as follows: forward, 5′‐ACGGACTGCAATACCAGAAAG‐3′; reverse, 5′‐CGTTCACACAGAGTGTCCCAG‐3′.

### 2.6 Western blots

Total protein was extracted from fresh tumor tissues and GIST cells, using the IP‐Lysate buffer (Beyotime, Shanghai), mixed by 1 × protease inhibitors (Selleck), and the concentration of protein was standardized by BCA protein detection. The total proteins (30 μg/lane) were separated on a 10% SDS‐PAGE gel and then transferred to NC membrane. After 1‐hour blocking in 5% fat‐free milk, the membrane, carrying separated proteins, was incubated with primary antibodies, anti‐DKK4 (1:250, Abcam, ab38589) and anti‐β‐actin (1:10 000, Cell Signaling Technology, #3700), at 4℃ overnight. After being bound with horseradish peroxidase (HRP)‐conjugated secondary antibody, the prepared membrane was imaged with ECL reagent solution (Share‐Bio, Shanghai).

### 2.7 Detection of serum DKK4 concentration

Individual peripheral blood samples, collected from 43 GIST patients, 20 healthy volunteers and 21 non‐GIST malignancies, were centrifuged to acquire the supernatant serum. Then the serum DKK4 concentration was detected using DKK4 ELISA kit (RayBiotech, America) according to its manufacturer's protocol. In addition, we compared the serum DKK4 concentration between tumor‐loading station with tumor‐free station of one patient.

### 2.8 Immunohistochemistry (IHC)

Prepared TMAs were put into xylene for deparaffinization and into gradient alcohol for rehydration, followed by antigen retrieval with citrate buffer. Then, their endogenous peroxidases were inactivated in 0.3% H_2_O_2_ at 37℃ for 20 minutes. After blocking in 10% bull serum albumin (BSA) for 1 hour, TMA was incubated with indicated primary antibodies at 4℃ overnight. Primary antibodies contain anti‐DKK4 (1:200 Abcam, ab38589), anti‐CD8A (1:400 Abcam, ab33786), and anti‐β‐catenin (1:500, Abcam, ab32572) antibodies. After combining with special HRP‐conjugated secondary antibody, the local proteins were marked with DAB substrate kit (Cell Signaling Technology) and the images were observed and captured by Nikon microscope. The profiles of local expression were described by four levels: negative as 0‐score, weak as 1, moderate as 2 and positive as 3, stratified by the staining intensity and positive area rates. Anti‐CD8A antibody was used for mark CD8^+^ T cells, and the mottling positive region combined with a miner nucleus was counted as one cell.

### 2.9 Cell Immunofluorescence

Pretreated cells were seeded on chamber slides (81201, ibidi), followed by being fixed with 4% paraformaldehyde, permeated with 0.2% Triton X‐100 (P0096, Beyotime) and blocked with 10% BSA. Then the slides were treated with primary antibodies at 37℃ for 1 hour. The primary antibodies contain anti‐DKK4 (1:100, Abcam, ab38589), anti‐c‐KIT (1:200, Abcam, ab111033) and anti‐β‐catenin (1:250, Abcam, ab32572) antibodies. After exhaustive soakage in PBS, they were incubated with corresponding secondary immunofluorescence antibodies at 37℃ for 1 hour. Digital images were taken using confocal microscopes, after counterstaining with DAPI.

### 2.10 Proliferation, growth, and migration assay of tumor cells

To assess the proliferative ability, tumor cells were seeded into 96‐well plates (1 × 10^4^ cells/well), then the cell viability was determined using Cell Counting Kit‐8 at the same interval, for 6 days continuously and the results were shown as proliferation curves.

To evaluate the growing ability of a single tumor cell, colony formation was performed in our study. Tumor cells were diluted to 3 × 10^3 ^cells/mL in 6‐well plates, cultured in the same conditions for 20 days, followed by being fixed with 4% paraformaldehyde and stained with 0.2% crystal violet. The colonies were counted by visual observation, which reflected the growing ability of a single cell.

The capacity of migration was estimated by transwell assay. About 2 × 10^4^ tumor cells, in 200 μL serum‐free medium, were put into the upper space of Boyden chamber (Millipore), with 700 μL 10% FBS medium in the bottom chambers. This system was incubated at 37℃ for 24 hours, waiting for cells’ migrating. Subsequently, the migrating cells were fixed with paraformaldehyde, stained with crystal violet, and counted under microscope.

### 2.11 Imatinib resistance assay

To assess the cells’ resistance to imatinib, half maximal inhibitory concentration (IC50) was adopted in our study. Tumor cells were seeded in 96‐well plates with the density of 95%, followed by treatment with gradient imatinib. After 3 days, the cell viability was detected using Cell Counting Kit‐8, and GraphPad 7.0 was used for calculation.

### 2.12 Chemotaxis assay of peripheral blood mononuclear cells (PBMCs)

PBMCs were separated from the peripheral blood samples of healthy volunteers, through gradient centrifugation with lymphocytes separating medium. Then the prepared PBMCs were put into the upper chamber of transwell assay, with the conditioned medium collected from DKK4‐NC and DKK4‐Sh1 GIST‐T1 cells in the bottom chambers. This system was incubated at 37℃ for 5 hours, followed by counting the migrating cells toward conditioned medium. All the cells in the bottom medium were marked with anti‐CD8A antibody, and counted by flow cytometry. So, the numbers of migrating CD8 + T cells could be compared among these three groups.

### 2.13 Immune cells co‐culturing assay

To verify the tumor‐inhibited extents of cytotoxic immune cells, pretreated tumor cells were co‐cultured with CD8^+^ T cells for 1 day, which were isolated from PBMCs. Then the tumor cells were harvested for cell apoptosis assay. Detecting samples were stained with propidium iodide (PI) and Annexin V‐FITC (BD Pharmingen), followed by being analyzed with flow cytometry.

### 2.14 Gene set enrichment analysis

Gene set enrichment analysis (GSEA) was conducted using the JAVA program as documented.^35^ GSEA is a computational method to search for particular gene sets corresponding with existing functional gene sets between two groups. Briefly, we performed GSEA on our expression profile data by differently grouping: high‐risk group vs low‐risk group, and DKK4 high‐expression group vs low‐expression group. Hallmarks gene set, KEGG gene set and Immunological Signatures gene set were involved in our analysis. The results were shown with the diagrams, labelled by normalized enrichment score (NES), false discovery rate (FDR) q‐value and *P*‐value.

### 2.15 Statistical analysis

The software, conducting statistical analysis in this study, contained SPSS 13.0 and GraphPad 7.0. Overall survival and recurrence‐free survival time of 132 patients were analyzed by Kaplan‐Meier plot method, and multivariate Cox regression analysis was based on the patients’ recurrence‐free survival. Differences between two groups were evaluated by two‐tailed Student's *t* test and Welch's *t* test. Correlation analyses were performed with Pearson correlation coefficients and nonparametric Spearman correlation coefficients. *P* < .05 was considered as statistically significant.

## 3 RESULTS

### 3.1 DKK4 is uniquely expressed in GISTs and universally upregulated in high‐risk samples

**Figure 1:**
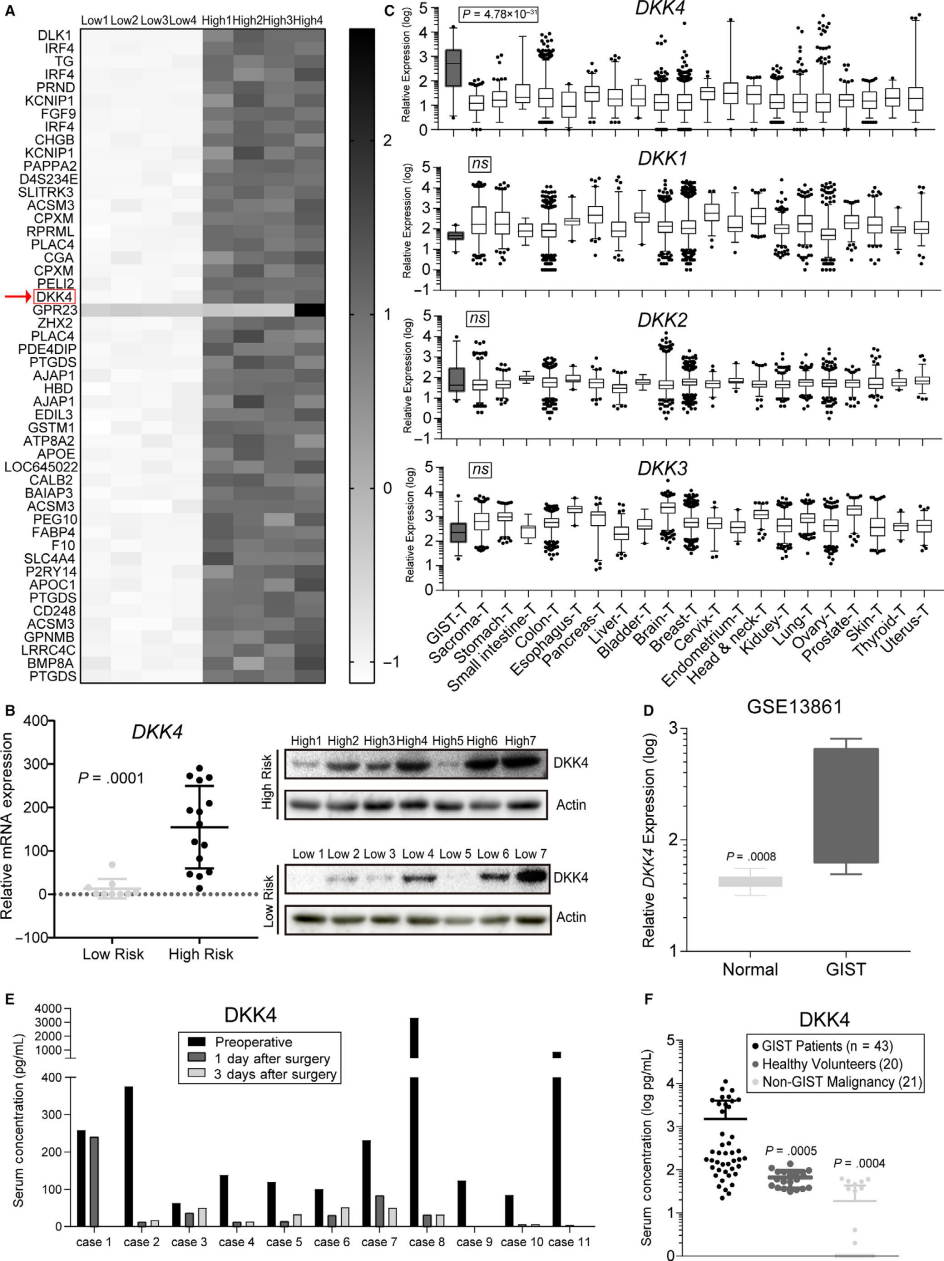
Expression patterns of *DKK4* in human gastrointestinal stromal tumor (GIST) specimens. A, Heatmap of expression profile analysis performed between low‐ and high‐risk GISTs, shown as the top 50 genes by fold‐change values. The red arrowhead points to *DKK4*. B, Validation of DKK4 overexpression with fresh GIST tissues, conducted in mRNA levels (left panel, Welch's *t* test is calculated for differences) and in protein levels (right panel). C, Patterns of DKK family's expression in GENT database (*P* value shows differences between GIST and the rest). D, Relative expressions of *DKK4* in GISTs (n = 6) and normal gastric tissues (n = 19) based on GSE13861 database. (E, F) Profiles of individual serum DKK4 concentration (E shows the alternations with tumor excised from body, and F shows the differences between GIST and healthy volunteers, non‐GIST malignancies, Welch's *t* tests are done with GIST group as control)

To explore distinct tumor biomarkers, well indicating the risk of GIST patients, we compared the expression profiles of freshly excised tumor tissues from four high‐risk and four low‐risk GISTs, stratified by modified National Institute of Health (NIH) consensus criteria.^36^, ^37^ Among those significantly upregulated genes (*P* < .05 and fold change > 4) in high‐risk samples, *DKK4*, as a canonical Wnt signaling inhibitor‐coding gene, came into our sights (Figure 1A). Then, we verified the aberrant accumulation of DKK4 in mRNA and protein levels within a bigger patients’ cohort (Figure 1B). These results both indicated that *DKK4* was upregulated in high‐risk GIST samples, compared with the low‐risk ones.

Multiple neoplastic data from GENT database^38^ showed that, the expression of *DKK4* in GIST was much higher than other various tumors (*P* = 4.78 × 10^−31^), containing sarcoma and digestive system neoplasms, which was unique from the other three members (Figure 1C). And the expression of *DKK4* was higher in GISTs than normal gastric tissues (Figure 1D). In view of DKK4 as a secretory protein, the profile of DKK4 concentration in individual peripheral blood was described. We found that serum DKK4 concentration was decreased to a large extent with the tumor excised from body (Figure 1E). In addition, serum DKK4 concentration from GIST patients was higher than healthy volunteers and non‐GIST malignancy patients (*P* = 0.0005 and 0.0004, respectively, Figure 1F), further supporting the discovery of unique expression of *DKK4* in GISTs. Taken together, these clinical data support that *DKK4* is uniquely activated in GISTs compared with other malignancies, and tend to be upregulated in high‐risk ones.

### 3.2 Aberrantly expressed DKK4 is correlated with poor prognosis in GIST patients

**Figure 2:**
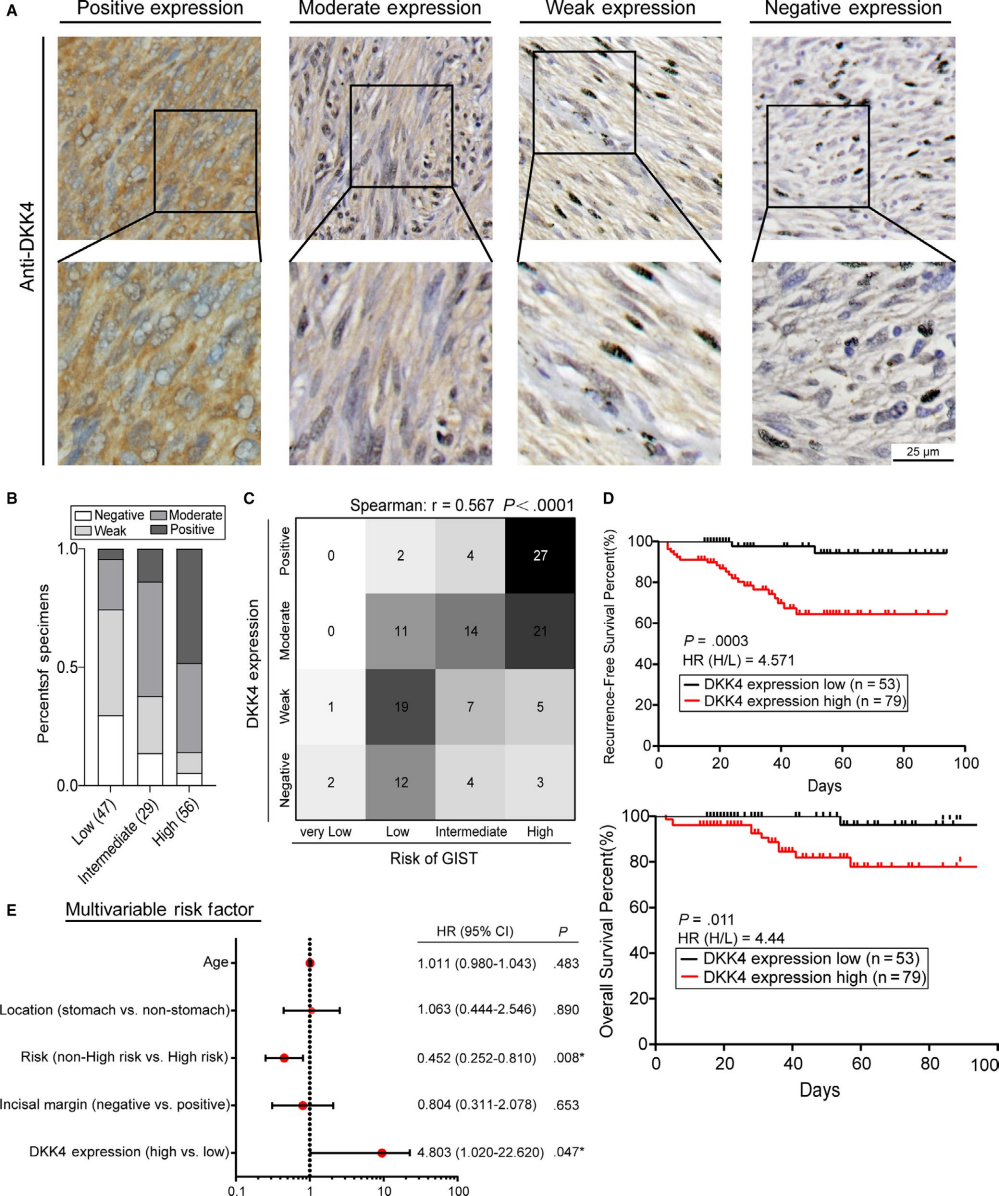
Prognostic values of DKK4 in human GIST specimens. A, Representative images of local DKK4 expression of various GIST samples, by IHC. B, Histograms illustrated the proportions of different DKK4 expressing levels in the low‐, intermediate‐ and high‐risk GISTs (n = 47, 29 and 56, respectively). C, Correlation between the local expressions of DKK4 and patients’ risk stratifications, shown by the heatmap and calculated by nonparametric Spearman coefficients (n = 132). D, Kaplan‐Meier analysis of the prognostic value of DKK4 expression based on recurrence‐free survival (RFS, upper panel) and overall survival (OS, lower panel). HR(H/L) means the hazard ratio of high‐expression group to low‐expression group. E, Multivariate Cox regression analyses based on RFS

To further confirm the event of aberrant *DKK4* overexpression in high‐risk GISTs, GIST tumor tissue microarray (TMA), containing 132 cases of GIST patients, was prepared for DKK4 detection in local microenvironment by IHC analysis. We found that DKK4 was specifically located around tumor cells region, indicating that it belongs to tumor‐derived secretory protein (Figure 2A). Corresponding with our previous findings, the majority of high‐risk GISTs overexpressed DKK4, which was much higher than the positive rate in low‐risk samples (85.7% and 27.7%, respectively, Figure 2B). Meanwhile, we found that the levels of local DKK4 expression were correlated with tumors’ size and mitosis (Table 1), and the expression level of DKK4 was positively correlated with the risk stratification for each patient (Spearman *r* = .567, Figure 2C). In addition, Kaplan‐Meier analysis suggested that overexpression of DKK4 predicted poor prognosis for both overall survival (OS) and recurrence‐free survival (RFS, Figure 2D). Owing to the appearance that DKK4 accumulation had less impairment on OS than RFS, we suspected OS was influenced by more factors than RFS for GIST patients. Then, we chose RFS as the prognosis indicator to conduct multivariate Cox regression analysis, pointing out high DKK4 expression and high‐risk stratification as independent predictors of GIST recurrence (Figure 2E). Given that *DKK4* is overexpressed in high‐risk GISTs and responsible for patients’ poor prognosis, we demonstrate that it potentiates to be an excellent tumor marker for predicting individual's survival and monitoring recurrence.

**Table 1:** Correlation between DKK4 expression and clinicopathologic factors

	Expression of DKK4		
Factors	High (n = 79)	Low (n = 53)	*P* value
Age (y)	58.67 ± 12.693	57.91 ± 12.835	0.738
Sex			
Male	45 (57.0%)	29 (54.7%)	0.799
Female	34 (43.0%)	24 (45.3%)	
Location			0.068
Stomach	37 (46.8%)	35 (66.0%)	
Small intestine	34 (43.0%)	16 (30.2%)	
Others	8 (10.1%)	2 (3.8%)	
Mitosis (/50 HPF^a^)			**0.014**
<5	49 (62.0%)	45 (84.9%)	
5‐10	7 (8.9%)	2 (3.8%)	
>10	23 (29.1%)	6 (11.3%)	
Tumor size (cm)			**<0.0001**
<2	0 (0.0%)	3 (5.7%)	
2‐5	20 (25.3%)	32 (60.4%)	
5‐10	37 (46.8%)	12 (22.6%)	
>10	22 (27.8%)	6 (11.3%)	
Risk^b^			**<0.0001**
Very low	0 (0.0%)	3 (5.7%)	
Low	13 (16.5%)	31 (58.5%)	
Intermediate	18 (22.8%)	11 (20.8%)	
High	48 (60.8%)	8 (15.1%)	
Incisal margin			0.138
Negative	55 (69.6%)	43 (81.1%)	
Positive	24 (30.4%)	10 (18.9%)	

Note

The values in bold type are those with statistical significance (*P* < .05).

a HPF is the abbreviation of high power field.

b The modified National Institute of Health (NIH) consensus criterion was adopted.

### 3.3 Separately deregulating DKK4 in vitro has little impact on GIST biological behavior

**Figure 3:**
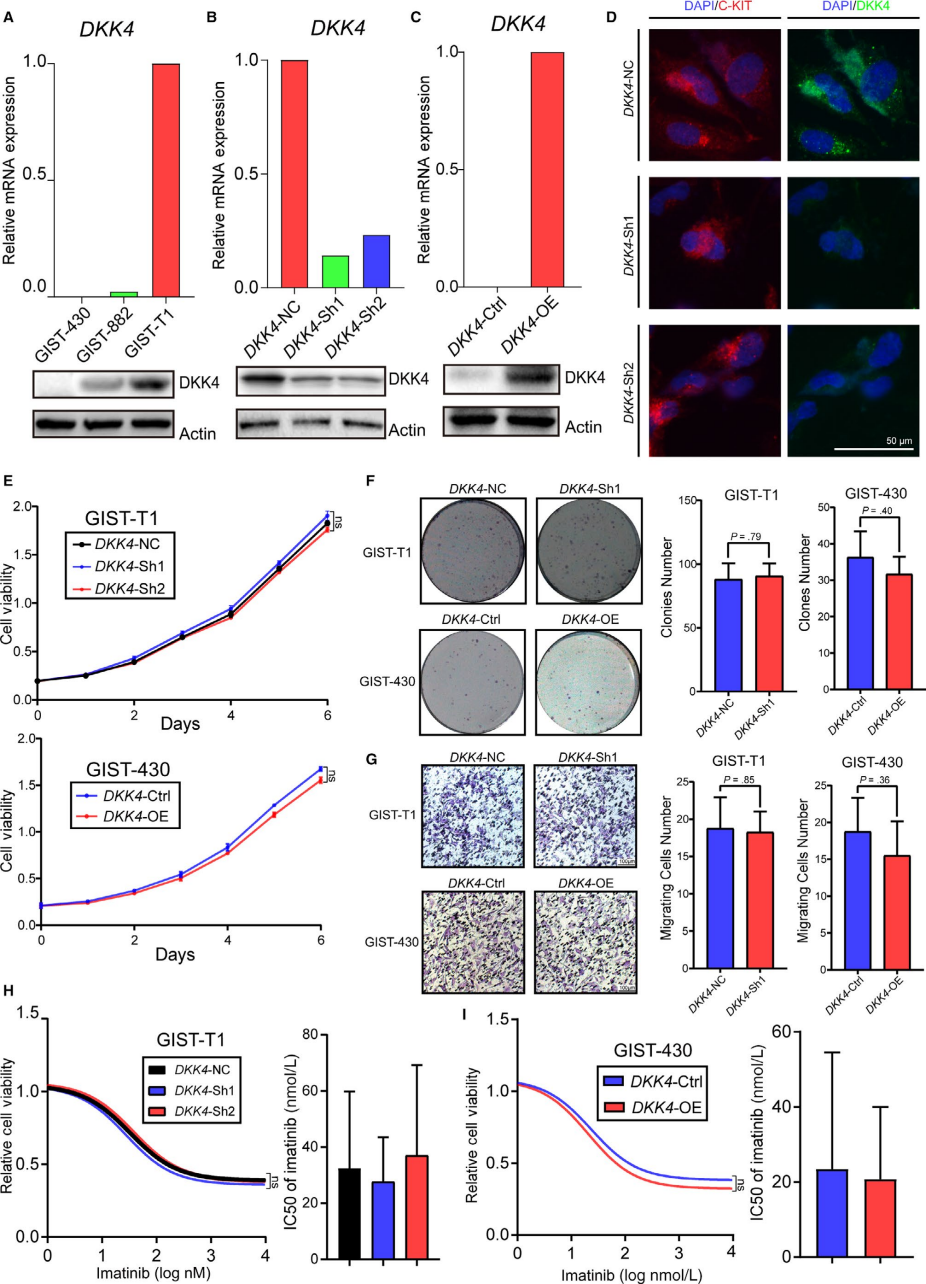
Little impact of deregulating *DKK4* in vitro on GIST biological behavior. A, Relative mRNA expression of *DKK4* by qPCR (upper panel), and protein levels by Western blot analysis (lower panel) in GIST cell lines. B, Knockdown efficiency of *DKK4* in GIST‐T1 cells in mRNA levels and protein levels. C, Overexpression efficiency of *DKK4* in GIST‐430 cells in mRNA levels and protein levels. D, Representative images of *DKK4* knockdown efficiency in GIST‐T1 cells, with positive reference as c‐KIT protein, by cell immunofluorescence. E, Proliferative potential of GIST‐T1 cells with *DKK4* knockdown (upper panel) and GIST‐430 cells with *DKK4* overexpression (lower panel), detected by CCK‐8 assay. F, Colony formation assay of GIST‐T1 cells with *DKK4* knockdown and GIST‐430 cells with *DKK4* overexpression. And colony numbers of tumor cells were shown as histograms. Student's *t* tests were performed for their statistical significances. G, Transwell assay of GIST‐T1 cells with *DKK4* knockdown and GIST‐430 cells with *DKK4* overexpression, scale bar: 100 μm and numbers of tumor cells migrating through the membrane are shown as histograms. Student's *t* tests were performed for their statistical significances. (H, I) Relative cell viability of GIST‐T1 and GIST‐430 cells treated with gradient imatinib for 72 h, which were indicated for tumor cell‐resistant ability to imatinib. Half maximal inhibitory concentrations (IC50) of GIST tumor cells to imatinib, combined with 95% confidence interval values were shown and histograms

Substantial clinical data, as mentioned above, suggested that *DKK4* served as a promoting tumor factor in GIST, which contradicted with some previous conclusions drawn in other tumors.^25^, ^39^ To investigate the concrete function of DKK4 in GIST, it was deregulated in GIST cell lines, and subsequent alternations were documented. *DKK4* expression profiles were shown in mRNA and protein levels of three cell lines we possessed (Figure 3A). In GIST‐T1 cell, with the highest expression level, *DKK4* was knocked down to about 80% extent (Figure 3B,D). It was strange that no significant alternations of cell proliferation, growth, migration, and drug resistance ability were seen in *DKK4*‐KD cells (Figure 3E‐H). Besides, we overexpressed *DKK4* in GIST‐430 cell, which held very low‐expression level, initially (Figure 3C). Unexpectedly, the *DKK4* overexpression should have a slight inhibitor effect on cell proliferation, growth, migration, and drug resistance ability, in despite of no statistical significance (Figure 3E‐G, I). Overall, those in vitro results indicate that separate knockdown or overexpression of *DKK4* has no or even opposite effects on GIST biological behavior as we discovered in patient samples and clinical events, which hints us of the underlying mechanism that DKK4 may paly promoting tumor role by targeting stromal cells not themselves in a paracrine way.

### 3.4 DKK4 overexpression is regulated by activated Wnt signaling

**Figure 4:**
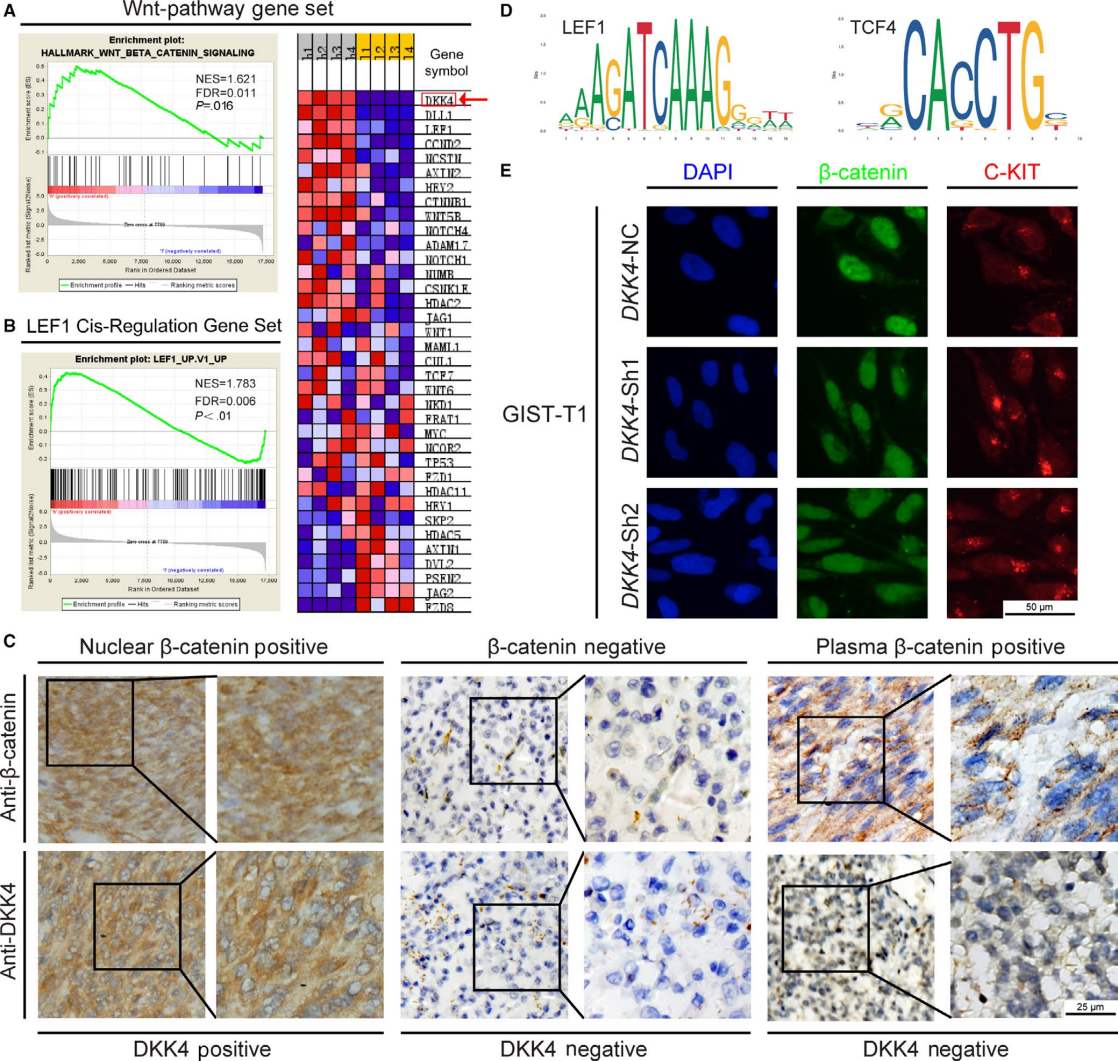
Upregulation of DKK4 regulating by activated Wnt pathway. (A, B) GSEA performed between high‐ and low‐risk GISTs, distinguished by Wnt pathway gene set (A, left panel) and LEF1 cis‐regulation gene set (B). DKK4 ranked as the top one of gene lists of Wnt pathway gene set. NES, normalized enrichment score; FDR, false discovery rate. C, Representative images of β‐catenin nuclear positive, β‐catenin negative and β‐catenin plasma positive specimens, with their corresponding profiles of DKK4 expression. D, Prediction of the transcriptional factors, LEF1 and TCF4, targeting at the promoter region of DKK4, using JASPAR website. E, The intracellular localization of β‐catenin with *DKK4* knockdown in GIST‐T1 cells, and c‐KIT served as the positive reference

To explore the underlying mechanism of promoting tumor behaviors of DKK4, our expression profile data were analyzed by GSEA software. When comparing high‐ with low‐risk GISTs, Wnt pathway gene set was enriched in high‐risk group, using the Hallmarks gene set, among which *DKK4* ranked as the top one corresponding gene (Figure 4A). What's more, *DKK4* also accorded to cis‐regulation gene set of *LEF1* (Figure 4B). In consideration of LEF1 and TCF4 as canonical Wnt pathway transcriptional factors (TFs), we speculated that *DKK4* was one of Wnt pathway target genes. And previous evidence had proved that Wnt signaling activated and played a pro‐progression role in advanced GIST. This was verified by IHC detection of DKK4 and β‐catenin, and TF binding sites prediction of LEF1 and TCF4 on the promoter region of *DKK4 *(Figure 4C,D). In addition, deregulation of DKK4 had no impact on the translocation of β‐catenin in an autocrine way (Figure 4E), which supported the assumption that DKK4 might interact with stromal cells in a paracrine way. Collectively, these findings indicate that *DKK4* overexpression is regulated by active Wnt pathway in high‐risk GISTs and it might play vital roles in a paracrine way.

### 3.5 DKK4 overexpression negatively correlates with cytotoxic immune cell activation

**Figure 5:**
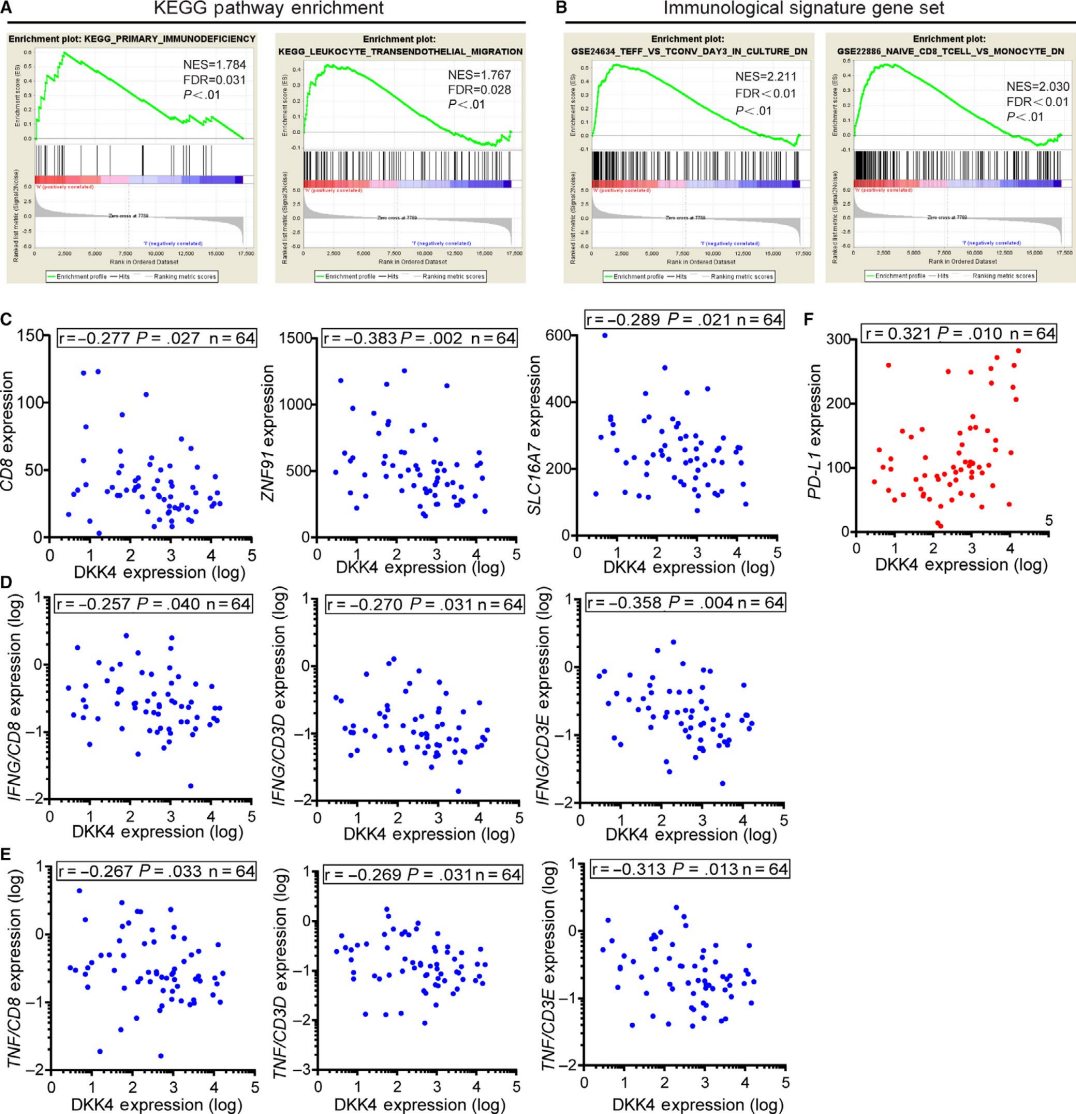
Negative correlations between the upregulated *DKK4* and cytotoxic immune cell activation. A, GSEA performed between high‐ and low‐ expression of *DKK4* group, using KEGG gene set. B, GSEA performed between *DKK4* high‐ and low‐ expression groups, using Immunological Signatures gene set. (C‐F) Correlation analyses between *DKK4* expression and canonical immunological markers with original data from GENT database, calculated by Pearson coefficients (n = 64). The blue scatter diagrams indicated negative correlation, while red scatters pointed to positive correlation

The eight GIST samples were divided into two groups by *DKK4* expression, followed by GSEA using Kyoto Encyclopedia of Genes and Genomes (KEGG) gene set. We took notice of the recurrent immune‐related gene sets, such as primary immunodeficiency and leukocyte transendothelial migration (Figure 5A), which reminded us of the immune microenvironment alternations during tumor progression. During next round analysis using immune‐related gene set, reduplicative results supported that *DKK4* high‐expression group was prone to impaired immune response groups (Figure 5B), which supposed the negative correlation between *DKK4* overexpression and cytotoxic immune cell activation.

To further confirm this assumption, original data from GENT website were accessed for correlation analysis. We showed the negative correlation between *DKK4*, derived from tumor cells, with the markers of CD8^+^ T cells, such as *CD8*, *ZNF91,* and *SLC16A7* (Figure 5C), indicating that *DKK4* overexpression decreased CD8^+^ T cells infiltrating into TME.^40^ What's more, interferon‐γ (IFN‐γ) and tumor necrosis factor (TNF), as typical effector cytokines of T cells, were standardized by T cells’ marker (CD3) for the assessment of cytotoxic immune cells activity, which showed consistent negative correlation with *DKK4* expression (Figure 5D,E). Interestingly, we found that the expression of *DKK4* was strongly positively correlated with the local expression of *PD‐L1* (Figure 5F), one of the proved immune checkpoint mechanisms in GIST. Taken together, these results support that local accumulation of DKK4 promotes the formation of immune suppressive microenvironment with low infiltrating immune cells, inactivation of cytotoxic immune cells and high expression of immune checkpoint molecules.

### 3.6 Tumor‐derived DKK4 inhibits cytotoxic immune cells infiltration and activation in TME

**Figure 6:**
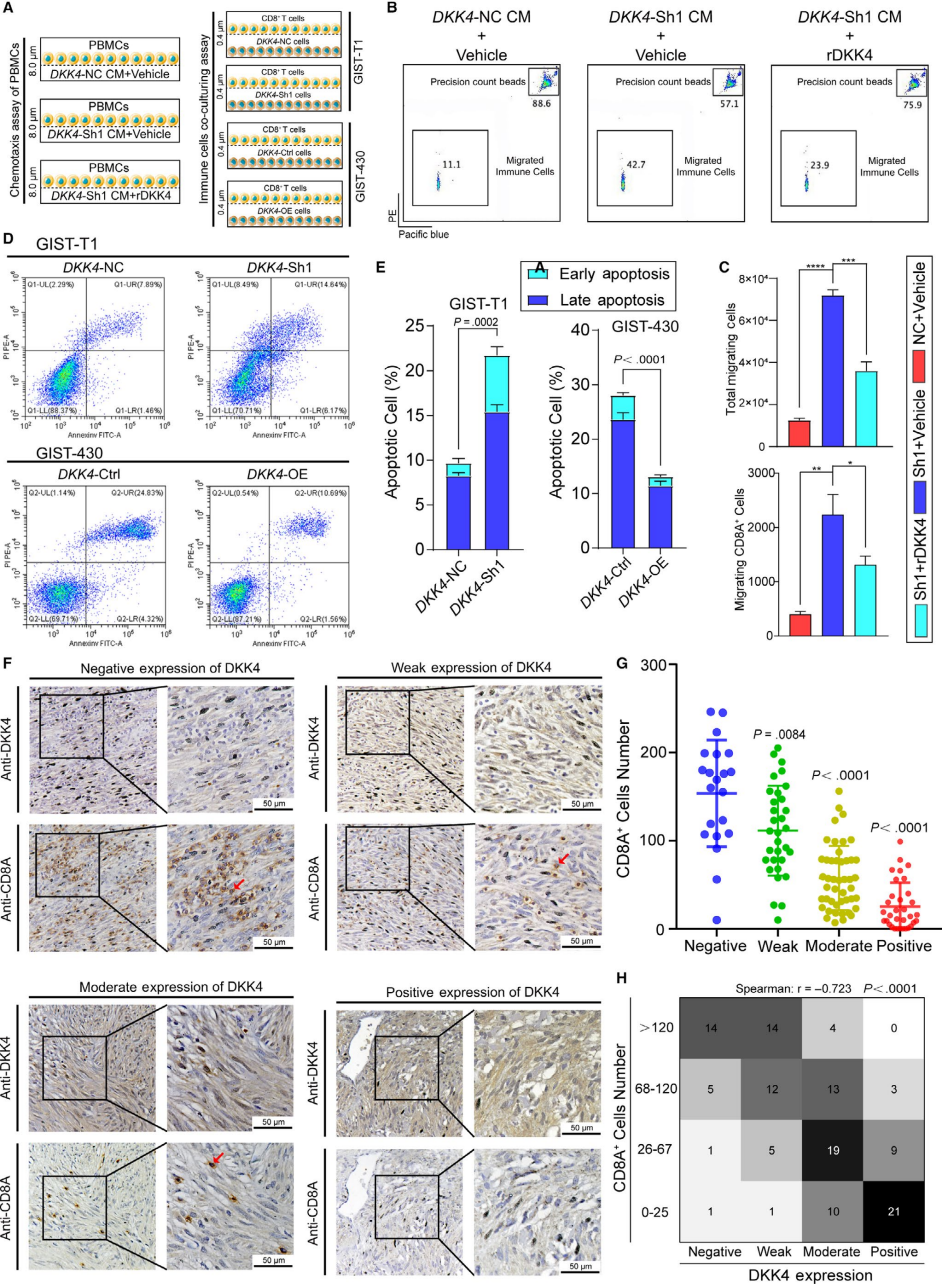
Tumor‐derived DKK4 decreased and weakened infiltrating immune cells in vitro and in human specimens. A, Schematic diagram of chemotaxis assay of PBMCs and immune cells co‐culturing assay. CM, conditioned medium. B, Chemotaxis assay of PBMCs, using the conditioned medium collected from DKK4‐NC and DKK4‐Sh1 GIST‐T1 cells, with or without exogenous DKK4 protein. C, The total migrating immune cells and migrating CD8^+^ cells were counted by flow cytometry and shown as histograms. *P < .05, **P < .01, ***P < .001, ****P < .0001. (D, E) Apoptosis rates of GIST‐T1 and −430 cells with CD8^+^ T cells existing, revealed by cell apoptosis assay. F, Representative images of local DKK4 expression and infiltrating CD8^+^ T cells. The red arrowheads point to CD8^+^ T cells. G, The CD8^+^ T cells were counted, corresponding to different levels of local DKK4 expression. Student's *t* tests were done with Negative group as control. H, Heatmap of individual local DKK4 expression and corresponding infiltrating CD8^+^ T cells, analyzed by Spearman coefficients

To validate our speculation that DKK4 inhibited cytotoxic immune cells migrating, peripheral blood mononuclear cells (PBMCs) were separated from healthy volunteer's peripheral blood, involved in the migration assay. We collected the supernate medium of GIST‐T1 cells, containing *DKK4*‐negative control (NC) and *DKK4*‐Sh1 cells, followed by conditioned culture with PBMCs, which took place in the transwell assay (Figure 6A, left panel). The numbers of migrated CD8^+^ T cells were significantly decreased in the direction to DKK4‐NC cells‐derived medium, compared with DKK4‐Sh1 cells‐derived medium. And recombinant DKK4 protein also decreased the migrating CD8^+^ T cells as the same (Figure 6B,C). This indicated that tumor cells‐derived DKK4 directly inhibited CD8^+^ T cells migrating, and this effect could be eliminated by *DKK4* knockdown. Subsequently, proved by the co‐culture apoptosis assay of tumor cells and CD8^+^ T cells (Figure 6A, right panel), deregulation of *DKK4* had a distinct impact on tumor cells’ biological behavior with stromal immune cells existing (Figure 6D,E).

To uncover the existence of this phenomenon in vivo, the profiles of local DKK4 expression and infiltrating CD8^+^ T cells were described in GIST, detected by IHC. Serial sections were detected by anti‐DKK4 and anti‐CD8A antibodies, respectively, for their correlation analysis (Figure 6F). With DKK4 expression scored and CD8A^+^ cells counted, we indicated that CD8^+^ T cells decreased significantly with the stronger expression of DKK4 (Figure 6G) and there was a negative correlation between them, which supported our previous findings in vitro (Spearman *r *= −.723, Figure 6H). Consistently, these data illustrated that tumor cells‐derived DKK4 decreased the infiltrating of cytotoxic immune cells to avoid antitumor immune response, which universally happened in humans.

## 4 DISCUSSION

Aberrant activation of Wnt pathway has been documented in advanced GIST previously.^19^, ^20^ However, the canonical Wnt signaling antagonist DKK4 appeared to be strongly correlated with patients’ risk stratification during our screening, which arouse our great interests. We have discovered that DKK4 was universally upregulated in high‐risk GIST samples. Tumors were stratified according to modified NIH consensus criteria, which defined the recurrence risk and predicted the individual prognosis after surgery.^41^ Conventional knowledge of DKK4’s biological function conflicted with our findings obtained from the tumor expression profile microarray. Then the analysis of TMA supported our discovery of aberrant DKK4 accumulation in high‐risk GIST, indicating that DKK4 overexpression was correlated with poor prognosis. In addition, multivariate Cox regression analysis recognized DKK4 high expression as an independent predictor of recurrence. All these clinical data supposed that DKK4 served as a promoting tumor factor in GIST. On the other hand, we speculate that DKK4 should be transcriptionally upregulated by activated Wnt pathway and serve as an effector of activated Wnt signaling driving malignancy in high‐risk GIST, owing to the facts from GSEA analysis, TFs prediction, IHC analysis, and previous studies (Figure 4).

The function of DKK4 in GIST was explored in vitro, subsequently. It was surprising that knockdown of DKK4 had little effect on biological behavior of GIST cell line, which reminded us of the possibility that DKK4 promoted tumor progress by contacting with stromal cells. Overexpression of DKK4 slightly abolished the malignancy of GIST‐430 cells, which is supposed to be that, redundant DKK4 in vitro could only target the tumor cells to inhibit their Wnt signaling, without an integrated coping mechanism for DKK4 accumulation and other TME stromal cells existing.

Hinted by previous studies and GSEA results, we focused on the relationship between DKK4 and local immune response.^34^ We noticed that the expression of *DKK4* was negatively correlated with cytotoxic immune cell activation, decreasing infiltrating immune cells and their secreted cytokines amounts, from the GENT database. Based on these analyses, we designed a chemotaxis assay of PBMCs, which demonstrated that tumor cells‐derived DKK4 could inhibit migration of cytotoxic immune cells. Subsequently, patients’ specimens were chosen as validation in vivo. Local DKK4 expression was strongly negatively correlated with CD8^+^ T cells existing in TME, supporting the inhibitor manner of DKK4 on infiltrating cytotoxic immune cells. Emerging evidence has shown that imatinib combined with immunotherapy produces a more significant effect on tumor suppression, because of the simultaneous effects that imatinib directly inhibits tumor cells and potentiates antitumor T cells response.^31^, ^32^, ^33^ DKK4, forming the immune‐suppressive microenvironment, is supposed to account for the weak sensitivity to imatinib to some extent, and is a potential therapeutic target to contribute to the combined therapy.

What's more, as a secretory protein, the profiles of DKK4 concentration in individual peripheral blood were described in our study. Consistent with findings elicited from tumor tissue, the inspiring results made it clear that DKK4 was a proper tumor biomarker for auxiliary diagnosis and recurrence monitor with individual peripheral blood samples.

Drug resistance and tumor recurrence are most of the concerns in clinical practices of GIST.^42^, ^43^ To deal with these, our study has revealed a novel tumor promoter, DKK4, which was upregulated by activated Wnt pathway in high‐risk GIST, promoting tumor progression via forming the immune suppressive microenvironment. Owing to necessity of immune cells participation in drug effects, DKK4 partially accounts for the weak sensitivity to RTK inhibitor in some cases, and can be recognized as a therapeutic target to enhance drug effects. In addition, the profiles of serum DKK4 concentration, combined with local tissue expression, are described as the individual prognosis predictor and recurrence monitor, which confers DKK4 the profound clinical values.

## CONFLICT OF INTEREST

None declared.

## AUTHOR CONTRIBUTIONS

Hui Cao and Zhi‐Gang Zhang designed the whole study scheme, and guided the experiment; Ming Wang and Bo Ni analyzed the experimental data and organized the manuscript. Chun Zhuang, Wen‐Yi Zhao and Lin Tu collected the clinical information. Bo Ni, Xin‐Li Ma, and Lin‐Xi Yang performed the cells experiments in vitro and validate our findings in vivo using patients' specimens.
